# Development of the Tilburg Pregnancy Distress Scale: the TPDS

**DOI:** 10.1186/1471-2393-11-80

**Published:** 2011-10-26

**Authors:** Victor JM Pop, Antoinette M Pommer, Monica Pop-Purceleanu, Hennie AA Wijnen, Veerle Bergink, Frans Pouwer

**Affiliations:** 1 Department of Medical Health Psychology, University of Tilburg, (Warandelaan), Tilburg, (Postbus 90153), The Netherlands; 2 Department of Psychiatry, Nijmegen MC, University Medical Centre, (Geert Grooteplein - Zuid), Nijmegen,(Postbus 9101), the Netherlands; 3 Department of Midwifery Science, AVM University, (Universiteitssingel), Maastricht, (Postbus 1256), the Netherlands; 4 Department of Psychiatry, Erasmus MC, University Medical Centre, ('s - Gravendijkwal), Rotterdam, (Postbus 2040), The Netherlands

## Abstract

**Background:**

Pregnant women with high levels of stress, depression and/or anxiety are at increased risk for adverse perinatal outcomes and impaired neurologic and emotional development of the offspring. Pregnancy specific instruments to measure psychological functioning during gestation are scarce and do not define items based on in-depth interviews of pregnant and recently delivered women. The current study developed a pregnancy specific scale that measures psychological functioning using in-depth interviews.

**Methods:**

Three focus groups were formed to discuss issues most relevant to pregnancy distress; 22 candidate items were derived for pilot testing (study I, n = 419) its psychometric properties by means of explorative factor analyses (EFA). This resulted in a 17-item TPDS which was further explored by confirmatory factor analyses (CFA) and concurrent and construct validity assessment (study II, n = 454).

**Results:**

EFA in study I suggested a two component solution (negative affect (NA) and partner involvement (PI)). CFA in study II resulted in a higher order model of the NA subscale into three more subscales: NA regarding confinement, delivery and general health. TPDS, EPDS and GAD-7 were all significantly correlated.

**Conclusions:**

The TPDS constitutes a valid and user friendly instrument to assess pregnancy distress. In addition to its proven ability to pick up pregnancy specific negative affect it also includes an important sub-scale measuring perceived partner involvement.

## Background

Stress, depression and/or anxiety during gestation not only have a major impact on women's health and quality of life, but also increases the risk of obstetrical complications (delayed fetal growth, preterm birth, low birth weight, increased technical interventions at delivery) [1-3], may affect infant (neuro- and emotional) development [4-7] and ultimately predict infant illness and health complaints [8]. In summary, women with a "higher vulnerability" profile (e.g., teenage pregnancy, low social-economic status, high levels of stress, depression and/or anxiety) seem to be at increased risk for adverse perinatal outcomes. With regard to mental health, there is thus a need to screen these vulnerable women with instruments designed to measure psychological functioning during gestation. Interestingly, such pregnancy specific instruments are scarce. As recently reviewed, the vast majority of measures used to assess psychological functioning in pregnant women to date were originally developed to detect depression and anxiety symptoms in general or during the postpartum period [9]. Apart from factors such as fearing for the baby's health and/or a painful delivery, specific stressors relevant to pregnant women remain unclear. The few scales that do target pregnancy specific symptoms of distress did not define items based on in-depth interviews of pregnant women, new mothers and health professionals [9]. There is one report of the development of a questionnaire after in-depth interviews in Pakistani pregnant women [10]. The Cambridge Worry Questionnaire was specifically developed to assess both the content and the degree of pregnant women's worries [11]. Although the nature of items was based on the experience of researchers with topics that were relevant to pregnant women, no structured in-depth interviews were used from the beginning and items were only related to potential worries of the pregnant women [11]. In addition there is also the Oxford 'worries about labour scale', however this scale was only validated in women who had already given birth [12].

To the best of our knowledge, the current study is the first attempt to develop and validate a pregnancy specific psychological functioning scale (i.e., the Tilburg Pregnancy Distress Scale: TPDS), taking into account the perspectives from (newly) mothers and clinicians.

## Methods

### Procedure

**Figure 1:**
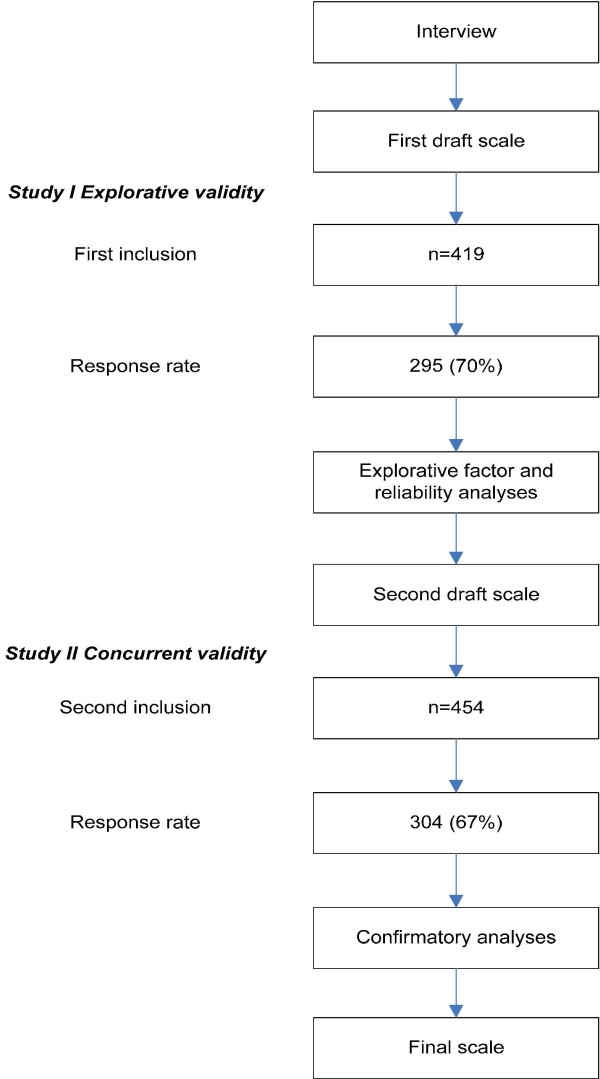
**Flowchart of the study**.

Prior to constructing the TPDS, three different focus groups were formed. The first group consisted of six midwives and six maternity nurses, the second group consisted of three primiparous and three multiparous pregnant women, and the third group consisted of six women who had recently delivered. Participants were encouraged to discuss issues which in their opinion were most relevant to pregnancy distress. All group interviews occurred under the supervision of a staff member of the Department of Clinical Health Psychology (University of Tilburg) and were recorded. The recorded texts were subsequently transcribed and evaluated by a broad-based expert panel (V.P., H.W., F.P., A.P.). The intention was to create a scale primarily based on the experiences of pregnant women since it is well known that psychological distress is often poorly recognized by clinicians [13]. Therefore no preliminary models were formulated and only double items were removed to construct the first draft of the questionnaire in order to lose as little information as possible. Based on the panel's consensus, a total of 22 items were derived from an original sample of 34 candidate questionnaire items for further pilot testing. To avoid a neutral response category the items were formatted on a four-point scale (ranging from 0 = "very often" to 3 = "rarely/never"). This version was subsequently distributed in 11 community midwife offices to examine its psychometric properties (study I, sample I). These analyses were then utilized to generate a more refined version of the TPDS, which was then distributed in 10 community midwife offices who did not participate in study I. Data from study II (sample II) were finally used to conduct a confirmatory factor analysis (CFA) and to determine the concurrent and construct validity (see Figure 1).

### Participants

Over a period of four months, 419 pregnant women visiting their midwife for antenatal care were invited to participate in the first pilot stage of the study, exclusion criteria were not being Caucasian and not being able to read Dutch sufficiently. Of these women, 295 (70%) consented to participate in study I (sample I). Subsequently, the second test version of the TPDS was distributed in another group of pregnant women with the same exclusion criteria used in sample I: 454 women were approached and 304 (67%) agreed to participate in study II (sample II). The women participating in each study had similar characteristics (Table 1).

**Table 1:** Characteristics of pregnant women in both study I (N = 295) and study II (N = 304)

	Group I					Group II				
		
Characteristics	N	%	Mean	SD	Range	n	%	Mean	SD	Range
*Demographics *										
Age										
Younger than 25	28	11,4				29	12,8			
26-30	94	38,4				112	36,8			
31-35	98	40,0				120	39,6			
Above 36	25	10,2				33	10,7			
Marital status										
With partner	245	100				298	98			
Education level										
Low	15	5				18	6			
Middle	136	46				131	43			
High	144	49				155	51			
Currently working outside the home	226	92,2				273	89,8			
*Obstetric history *										
Parity										
Primiparous	131	44,4				135	44,4			
Multiparous	164	55,6				169	55,6			
Previous abortion	63	25,7				88	28,9			
Term of gestation in weeks			26,56	9,45	6 -41			28,61	9,12	6 -41
*Life style *										
Any alcohol intake	19	7,7				21	6,9			
Any smoking	15	6,1				20	6,6			

The study was approved by the Medical Ethics Committee of the Máxima Medical Centre in Eindhoven/Veldhoven, the Netherlands.

### Measurements

#### Study I

The assessments for study I (sample I) consisted of the first pilot version of the TPDS and several customized questions regarding demographics (age, marital status, work, education, socio-economic status), obstetric history (parity, previous abortion, term of gestation, complications during current pregnancy and location of delivery) and lifestyle (alcohol use, smoking habits).

#### Study II

The assessment package for study II (sample II) comprised the second version of the TPDS, the Generalized Anxiety Disorder Anxiety Scale (GAD-7), the Edinburgh Postnatal Depression Scale (EPDS) and questions regarding demographics, obstetric history and life style. Furthermore, open ended questions were added enquiring about a previous diagnosis of depression or anxiety made by a general practitioner, psychiatrist or psychologist.

#### Generalized Anxiety Disorder Anxiety Scale (GAD-7)

The GAD-7 is a valid and reliable (α = 0.89) device for screening generalized anxiety disorder and for assessing its severity [14]. The GAD-7 consists of 7 items with response options ranging from 'not at all' to 'nearly every day'. Cut-off points of 5, 10, and 15 are associated with respectively mild, moderate, and severe levels of anxiety [15]. In the current study a cut-off of 10 was used to define high anxiety scores.

#### Edinburgh Postnatal Depression Scale (EPDS)

The Dutch version of the EPDS has been validated among postpartum women in the Netherlands and revealed appropriate psychometric characteristics with an α-coefficient of 0.87 [16]; it consists of ten items [17]. The total score ranges from 0 to 30, with higher scores indicating more depressive symptoms. Recently, the EPDS has also been validated in pregnant women [18]. In the current study, a cut-off of 12 was used to define high depression scores.

### Statistical methods

Statistical analyses were performed using the Statistical Package for Social Sciences (SPSS version 18.0, IBM, Chicago, Illinois, USA). The confirmatory factor analysis was done using AMOS (version 18, IBM, Chicago, Illinois, USA) and Lisrel 8.8.

#### Study I

First, exploratory factor analysis (EFA) was performed on the full 22-item pilot version of the TPDS. A principal component analysis with an oblimin rotation and a scree test were used to select factors for retention. The cutoff for item factor loadings was set at a coefficient level of ≥0.40. Internal consistency was measured by Cronbach's alpha for the total scale and for the possible subscales derived from factor analysis.

#### Study II

##### Confirmatory factor analysis

Confirmatory factor analysis (CFA) was used to test the stability of the factor structures found with EFA in study I (sample I). The comparative fit index (CFI), normed fit index (NFI), standardized root mean square residual (SRMR), and the root mean square error of approximation (RMSEA) were used to evaluate model fit. A CFI of ≥ 0.80 in combination with a NFI of ≥ 0.80 and a RMSEA of ≤ 0.06 are generally considered as indicators of adequate fit of a model [19]. A SRMR of less than 0.08 is generally considered as a good fit of the model [20]. The 'Estimate means and intercepts' option was selected to control for missing values.

##### Concurrent validity

Concurrent validity of the newly developed TPDS was tested by correlating (Pearson correlations, two-tailed) the TPDS and its possible subscales with the GAD-7 and the EPDS.

##### Construct validity

Until now, there is no psychiatric interview to diagnose pregnancy specific distress. Instead, construct validity was examined using a previous self-reported diagnosis of depression and/or anxiety as a proxy. Scores on the TPDS, the EPDS, and the GAD-7 between women with and without a previous history of depression/and or anxiety were compared. Single logistic regression (O.R; 95% CI) analyses were performed with a high score on the EPDS, the GAD-7 and the TPDS as dependent variables and a previous diagnosis of depression and/or anxiety as independent variable.

## Results

### Study I: explorative factor analysis with Oblimin rotation

All assumptions for conducting principal components analysis were met. The Kaiser-Meyer-Oklin value was greater than 0.60 (0.81) and the Bartlett's test of sphericity value was significant (*p *< 0.001). Although the un-rotated principle components analysis suggested a four factor model, Catell's scree test clearly suggested a two component solution. After oblimin rotation, a two factor model explaining 34% of the variance was revealed: a 'negative affect' factor (TPDS-NA), and a 'partner involvement' factor (TPDS-PI, Table 2). Six items that did not have a loading above 0.40 on either factor were deleted from subsequent analyses. Closer examination of these items revealed that some items were to general and others often yielded the same answers in most women. The resulting 16 item TPDS scale showed good internal consistency overall (α = 0.78), as well as for each subscale (TPDS-NA, 11 items, α = 0.80; TPDS-PI, 5 items, α = 0.80).

**Table 2:** Two factor solution from factor analysis with oblimin rotation in 295 pregnant women who completed the 22-item Tilburg Pregnancy Distress Scale; the Pattern Matrix

	Factor I Negative Affect	Factor II Partner Involvement
	
Eigenvalue	4.8	2.66
**Percentage of variance explained **	**21.64 **	**12.01**

1. enjoying pregnancy		.56
2. experience pregnancy together		.86
3. worry about delivery	.72	
4. partner and I closer together		.67
5. worry about pregnancy	.51	
6. worry about health of baby	.53	
7. concerns about job	.48	
8. feeling supported by partner		.80
9. concerns about finances	.48	
10. high workload		
11. insecure about qualities		
12. feeling beautiful		
13. fear of taking care of baby alone		
14. fear losing self-control	.59	
15. feeling angry		
16. choices concerning delivery	.50	
17. worry about delivery	.78	
18. feeling tense	.72	
19. sharing feelings with partner		.78
20. worry about gaining weight	.49	
21. concerned with physical discomforts	.49	
22. disliking pregnant belly		

A cut-off score of item loading of .40 was used and a minimum difference of .20 if an item had two loadings. For full text of items see appendix.

### Study II

#### Confirmatory factor analysis

**Figure 2:**
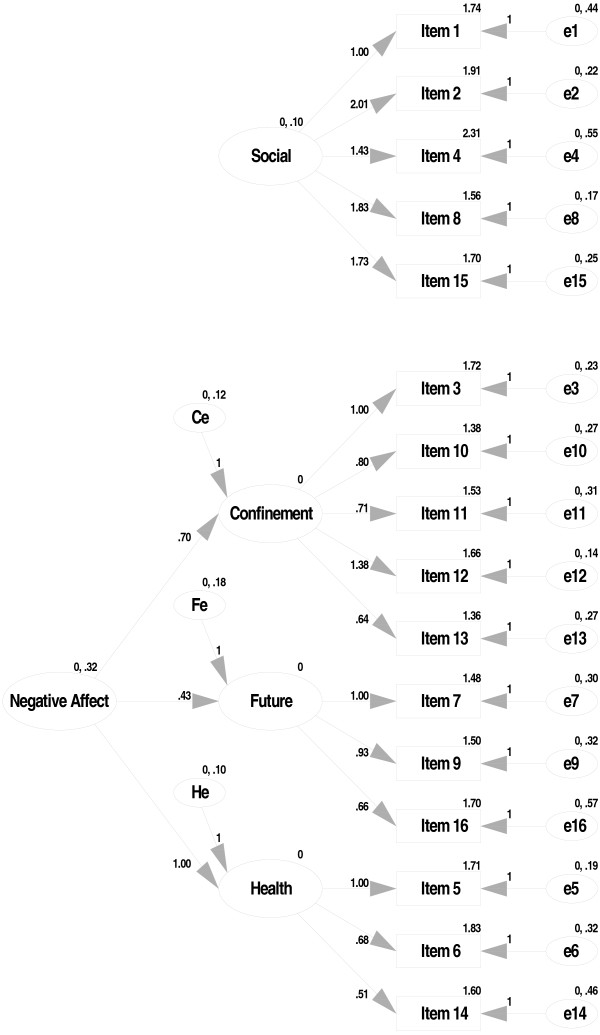
**Best fit model of the TPDS**.

Testing the above-mentioned simple structure, using CFA in a new sample of pregnant women revealed an inadequate fit: CFI's and NFI's < 0.80, and RMSEA's > 0.06. In addition, several error terms appeared to be correlated. Consequently, an additional analysis was conducted to explore higher order models. The resulting model had two main components: 'partner involvement' and 'negative affect', with the latter component having three subcomponents (negative affect with regard to confinement, with regard to the postpartum period, and with regard to general health) (Figure 2). This final model showed an adequate fit (CFI = 0.91, NFI = 0.83, SRMR = 0.07, RMSEA = 0.06). The two main subscales appeared to have good reliability (TPDS-PI, α = 0.80; TPDS NA, α = 0.81) (Table 3).

**Table 3:** Means, ranges and reliability scores of 304 pregnant women in study II on the 16-items Tilburg Pregnancy Distress Scale (TPDS), its subscales negative affect (NA) and partner involvement (PI) and the EPDS and the GAD-7

	No. of items	Range	M(SD)	Cronbach's alpha
**Total TPDS scale **	16	0-37	10.67 (5.81)	.78
**TPDS PI **	5	0-14	4.20 (2.90)	.80
**TPDS NA **	11	0-23	6.46 (4.70)	.81
**EPDS **	10	0-25	5.36 (4.33)	.84
**GAD-7 **	7	0-21	4.28 (3.69)	.86

#### Concurrent validity

As shown in Table 4, the TPDS and its subscales were significantly (inter) correlated with the EDPS and GAD-7 (*p's *< 0.05).

**Table 4:** Correlation matrix.

	TPDS	TPDS PI	TPDS NA	EPDS	GAD7
**TPDS **	1.00	.60**	.87**	.56**	.56**
**TPDS PI **	-	1.00	.12	.32**	.27**
**TPDS NA **	-	-	1.00	.49**	.52**
**EPDS **	-	-	-	1.00	.75**
**GAD7 **	-	-	-	-	1.00

Construct validity of the TPDS, its sub-scales in relation to the EPDS and GAD-7

**Correlation is significant at the 0.01 level (2-tailed)/* Correlation is significant at the 0.05 level (2-tailed)

#### Prevalence of anxiety and depression symptoms

Twenty-five women (8.2%) met the EPDS cutoff (> 11) for depression, and 26 women (8.6%) met the cutoff for high anxiety on the GAD-7 (>9). In the current study, these cut-offs corresponded with the 92^th ^and 91^th ^percentile scores on the EPDS and the GAD-7, respectively. Therefore, the cutoff of a high score on the TPDS and its subscales (= distressed woman) was set at the 90^th ^percentile which resulted in the following cutoff scores: for the overall scale > 17, for its sub-scale 'NA' > 12, and for its subscale 'PI' (> 7).

#### Construct validity

In total, 42 (13.8%) women reported a diagnosis of depression and/or anxiety before becoming pregnant of which four women reported both. Single logistic regression analyses revealed that a previous diagnosis was significantly related to a high level of depression (EPDS, O.R: 5.1, 95% CI: 2.1 - 12.4) and to heightened anxiety (GAD-7, O.R.: 8.6, 95% CI: 3.2 - 12.3) during gestation. A series of single logistic regressions showed that women with a previous diagnosis of depression and/or anxiety were also at increased risk for scores over the 90^th ^percentile on the overall TPDS (O.R. 2.8, 95% CI: 1.1 - 7.4), and the NA sub-scale of the TPDS (O.R. 2.8, (95% CI: 1.1 - 7.0). This was, however, not the case for the TPDS-PI sub-scale (O.R. .71, 95% CI: 0.2 - 2.2).

## Discussion

The aim of the current study was to develop a pregnancy specific psychological functioning scale. Based on the outcome of group interviews with pregnant women, new mothers and clinicians, a 16-item self-rating scale was developed (i.e., the TPDS; for full scale see appendix). Subsequent analyses confirmed that the TPDS has a two factor structure: 'negative affect' and 'partner involvement'. Negative affect, in turn, appeared to have three sub components: negative affect with regard to confinement, postpartum period and general health. Both AMOS and Lisrel showed appropriate structure of the final scale using CFA.

Although the overall TPDS and its NA sub-scale were only moderately correlated with well-recognized measures of depression (EPDS) and anxiety (GAD-7), indicating that the TPDS and its subscale 'NA' also assessed dimensions other than depression and anxiety, encouraging construct validity features may be derived from our finding that women with a previous diagnosis of depression/anxiety were at high risk for developing depressive and/or anxiety symptoms during pregnancy.

Interestingly, the current TPDS analyses indicated that perceived partner involvement (TPDS-PI sub-scale) constitutes a critically important variable for women during and after pregnancy (items 2, 4, 8, 16). The TPDS-PI sub-scale was only marginally correlated (r = .15) with the TPDS-NA sub-scale. Moreover, high scores on the TPDS-PI sub-scale were not related to a previous episode of depression/anxiety. Future research should concentrate on the impact of the woman's perception of little partner involvement during pregnancy. However, since partner involvement spontaneously emerged during the interviews these findings suggests that the TPDS-PI sub-scale constitutes a distinct dimension relevant to pregnant women.

To our knowledge the current paper is among the first to report on a pregnancy specific psychological functioning scale which was developed in close interaction with pregnant women, recently delivering mothers and health professionals for obstetric care. While over the last years there have been several attempts to develop pregnancy specific distress scales, most of these scales were adaptations from existing general depression and anxiety questionnaires [9]. Moreover, none of these scales were developed after in-depth interviews with pregnant women and clinicians [9]. The NA subscale of the TPDS has several items which are similar to the Cambridge Worry Questionnaire [11]. The correlations in the current study between the NA-subscale and the EPDS and the GAD-7 scales were also found in the validation study of the Cambridge Worry Scale [11]. The latter however, does not contain a sub-scale which specifically refers to the woman's perception of partner involvement.

Several recent studies have reported a relationship between high maternal distress levels during gestation and poor developmental outcomes in offspring [4-7]. Evidently, low perceived partner involvement adds to the pregnancy stress experienced by women and, as such, constitutes an important topic for future research. Likewise, poor parental relationships should be included in future studies as marital distress also constitutes a major threat to developmental outcome [21]. In view of this, one may also speculate whether those women who perceive poor partner involvement during gestation, are also at risk for continued poor partner interaction in the postpartum period.

The current study's key strength relates to the fact that the TPDS' first version originated from direct consultation with pregnant women, new mothers and clinicians. Other strengths include its large sample size, as well as the fact that the validity of the newly developed scale was examined in a separate cohort of women.

Limitations of the study include the fact that the participating women were all Caucasian, and that the term of gestation at which the women were assessed varied between 12 and 40 weeks. One may argue that the scores on the TPDS are trimester specific which would in turn call for future research to validate the TPDS per trimester.

However, as far as the NA-subscale is concerned, the Cambridge Worry Scale scores at different trimesters showed to be highly inter-correlated with appropriate validity at each trimester [11].

Not having a psychiatric interview to diagnose pregnancy specific distress is another limitation of the study. However such an interview doesn't exist yet, indicating the need for further research on this topic.

The clinical relevance of a pregnancy specific distress scale is that it allows for quick screening and, if needed, quick subsequent intervention with active partner participation when necessary.

## Conclusions

The current findings suggest that the TPDS constitutes a valid and user friendly instrument to assess pregnancy distress. In addition to its proven ability to pick up negative emotions regarding confinement, delivery and general health, the TPDS also includes an important sub-scale measuring perceived partner involvement. Future research should further elucidate the validity and use of the TPDS in clinical practice.

## List of abbreviations

TPDS: Tilburg Pregnancy Distress Scale; EFA: Explorative Factor Analyses; CFA: Confirmatory Factor Analyses; NA: Negative Affect; PI: Partner Involvement; EPDS: Edinburgh Postnatal Depression Scale; GAD-7: Generalized Anxiety Disorder Scale; CFI: Comparative Fit Index; NFI: Normed Fit Index; RMSEA: Root Mean Square Error of Approximation; SRMR: Standardized Root Mean square Residual.

## Competing interests

The authors declare that they have no competing interests.

## Authors' contributions

All authors have been involved with data collection, analyses, writing, or combinations of these activities and have approved the final manuscript.

## Pre-publication history

The pre-publication history for this paper can be accessed here:

http://www.biomedcentral.com/1471-2393/11/80/prepub

## Supplementary Material

Additional file 1 **Appendix**. The Tilburg Pregnancy Distress Scale. Click here for file
