# Supplementary material for: Development of the Tilburg Pregnancy Distress Scale: the TPDS
Source: BMC Pregnancy Childbirth. 2011 Oct 26;11:80. doi: 10.1186/1471-2393-11-80 (PMC3216243; doi:10.1186/1471-2393-11-80)
Supplement: Additional file 1 — Appendix. The Tilburg Pregnancy Distress Scale. [file 1471-2393-11-80-S1.PDF]

APPENDIX

Tilburg Pregnancy Distress Scale

The following questions relate to the way you perceive your pregnancy. **Circle** the **box** that best reflects how you felt during **the last 7 days**. Please circle only one answer to each question

		**Very often**	**Fairly often**	**Now and then**	**Rarely or never**
1.	I am enjoying my pregnancy	☐	☐	☐	☐
2.	I feel like my partner and I are enjoying the pregnancy together	☐	☐	☐	☐
3.	I worry about the pregnancy	☐	☐	☐	☐
4.	The pregnancy has brought my partner and I closer together	☐	☐	☐	☐
5.	I worry about the delivery	☐	☐	☐	☐
6.	I worry about the health of my baby	☐	☐	☐	☐
7.	I worry about my job once the baby is born	☐	☐	☐	☐
8.	I feel supported by my partner	☐	☐	☐	☐
9.	I worry about our financial situation after childbirth	☐	☐	☐	☐
10.	I am afraid I will lose self-control during delivery	☐	☐	☐	☐
11.	I often think about choices concerning the delivery	☐	☐	☐	☐
12.	The delivery is troubling me	☐	☐	☐	☐
13.	I get very tense hearing stories about deliveries	☐	☐	☐	☐
14.	I am concerned that the physical discomforts of pregnancy might persist after childbirth	☐	☐	☐	☐
15.	I can really share my feelings with my partner	☐	☐	☐	☐
16.	I worry about gaining too much weight	☐	☐	☐	☐

Key to calculate scores

Item : 3, 5, 6, 7, 9, 10, 11, 12, 13, 14 and 16 should be recoded (3=0, 2=1, 1=2, 0=3).
